# The Pneumonia-Coccus of Friedlander

**Published:** 1885-10

**Authors:** 


### Selections.

### The Pneumonia-Coccus of Friedlander.

Dr. George M. Sternberg, U. S. A., in a paper which appears in the July number of *The American Journal of the Medical Sciences*, calls attention to the so-called pneumonia-coccus of Friedlander, which he claims is in fact identical, specifically, with a micrococcus which he previously described, and which is found in normal human saliva, and with that found by Pasteur in the blood of rabbits which had been injected with the saliva of a child which died of hydrophobia in one of the Paris hospitals. This micrococcus he names *Micrococcus-Pasteuri*. The capsule, or mucous envelope, which sometimes surrounds this micrococcus, described by Friedlander in 1883, and photographed by Sternberg two years previously, cannot be accepted as a distinguishing character of this species, inasmuch as it is not constantly present, and the circumstances upon which its development depends have not been accurately determined. It is established that this is a pathogenic organism, as far as certain lower animals are concerned, and that its pathogenic power varies under different circumstances. It seems extremely probable that this micrococcus is concerned in the etiology of croupous pneumonia, and that the infectious nature of this disease is due to its presence in the fibrinous exudate into the pulmonary alveoli.

But this cannot be considered as definitely established by the experiments which have thus far been made on lower animals. The constant presence of this micrococcus in the buccal secretions of healthy persons indicates that some other factor is required for the development of an attack of pneumonia; and it seems probable that this other factor acts by reducing the vital resisting power of the pulmonary tissues, and thus making them vulnerable to the attacks of the microbe. This supposition enables us to account for the development of the numerous cases of pneumonia which cannot be traced to infection from without. The germ being always present, auto-infection is liable to occur whenever from alcoholism, sewer-gas poisoning, crowd-poisoning, or any other depressing agency, the vitality of the tissues is reduced below the resisting point. We may suppose also that a reflex vaso-motor paralysis, affecting a single lobe of the lung, for example, and induced by exposure to cold, may so reduce the resisting power of the pulmonary tissue as to permit this micrococcus to produce its characteristic effects.

Again, we may suppose that a person whose vital resisting power is reduced by any of the causes mentioned, may be attacked by pneumonia from external infection with material containing a pathogenic variety of this micrococcus, having a potency, permanent or acquired, greater than that possessed by the same organism in normal buccal secretions.

